# Functional Metagenomic Investigations of Microbial Communities in a Shallow-Sea Hydrothermal System

**DOI:** 10.1371/journal.pone.0072958

**Published:** 2013-08-06

**Authors:** Kai Tang, Keshao Liu, Nianzhi Jiao, Yao Zhang, Chen-Tung Arthur Chen

**Affiliations:** 1 State Key Laboratory of Marine Environmental Science, Xiamen University, Xiamen, Fujian, China; 2 Institute of Marine Microbes and Ecospheres, Xiamen University, Xiamen, Fujian, China; 3 Institute of Marine Geology and Chemistry, National Sun Yat-sen University, Kaohsiung, Taiwan, Republic of China; University of Illinois, United States of America

## Abstract

Little is known about the functional capability of microbial communities in shallow-sea hydrothermal systems (water depth of <200 m). This study analyzed two high-throughput pyrosequencing metagenomic datasets from the vent and the surface water in the shallow-sea hydrothermal system offshore NE Taiwan. This system exhibited distinct geochemical parameters. Metagenomic data revealed that the vent and the surface water were predominated by *Epsilonproteobacteria* (*Nautiliales*-like organisms) and *Gammaproteobacteria* ( *Thiomicrospira* -like organisms), respectively. A significant difference in microbial carbon fixation and sulfur metabolism was found between the vent and the surface water. The chemoautotrophic microorganisms in the vent and in the surface water might possess the reverse tricarboxylic acid cycle and the Calvin−Bassham−Benson cycle for carbon fixation in response to carbon dioxide highly enriched in the environment, which is possibly fueled by geochemical energy with sulfur and hydrogen. Comparative analyses of metagenomes showed that the shallow-sea metagenomes contained some genes similar to those present in other extreme environments. This study may serve as a basis for deeply understanding the genetic network and functional capability of the microbial members of shallow-sea hydrothermal systems.

## Introduction

The discovery of deep-sea hydrothermal vents in the late 1970s expanded our knowledge of the extent of microhabitats for microorganisms and the possible origins of life on Earth [1,2]. Deep-sea hydrothermal vent chimneys are known to harbor numerous metabolically diverse microorganisms along sharp physical and chemical gradients [2–6]. The geochemistry and microbial communities of deep-sea hydrothermal systems, such as the Lost City chimneys in the Mid-Atlantic Ridge [3–5] and *Alvinellapompejana* , a polychaete in the East Pacific Rise hydrothermal vent fields [6], have been studied in significant detail. Shallow-sea hydrothermal vents (at water depths of <200 m) are far more ubiquitous than previously recognized [7,8]. They provide more instant access to investigate the metabolic potential and adaptation of microbial communities to extreme environments. However, comparatively few investigations of microbial communities in shallow-sea hydrothermal fields have been conducted.

Shallow-sea hydrothermal vents usually occur near active coastal or submarine volcanoes [7,8]. Such an area of andesite-hosted shallow-sea hydrothermal venting at pH ~1.5−5.0 (acidic) and 30 °C to 116 °C with slightly sulfidic fluids is located 1 km east of Kueishantao Island, near the southern end of the Okinawa Trough [9,10]. Elemental sulfur (S^0^) is naturally enriched in shallow-sea hydrothermal fluids [9], and gas discharging from these vents is dominated by carbon dioxide (CO_2_). Among previously investigated hydrothermal fields [11], fluids in the shallow-sea hydrothermal system near Kueishantao Island contain moderate amounts of hydrogen (H_2_) and methane (CH_4_) but very low amount of hydrogen sulfide (H_2_S) because of short interactions of rock and seawater [9]. By contrast, fluids at the serpentinite-hosted Lost City hydrothermal field are highly enriched in CH_4_ and H_2_ with near-zero concentrations of CO_2_. In addition, the Lost City chimneys are alkaline (pH ~9.0 to 11.0) and contain more abundant H_2_S than the shallow-sea vents [11]. Deep-sea vent communities are often dominated by symbiotrophic forms, such as microbial communities associated with *Alvinellapompejana* [6] and *Riftiapachyptila* [12]. The geochemical conditions of *A* *. pompejana* are characterized by venting of fluids at 29 °C to 84 °C and pH ~5.3 to 6.9 (slightly acidic to near neutral) with a similar level of free H_2_S as the shallow-sea vents [6].

With the development of next-generation sequencing technologies, tag pyrosequencing of 16S rRNA genes has been applied to reveal a new and enormous bacterial diversity in deep-sea or shallow-sea hydrothermal environments [3,4,13]. For example, serpentinite-hosted Lost City chimneys harbor endosymbionts with close phylogenetic relationships to the sulfur-oxidizing bacterium *Thiomicrospira* of the class *Gammaproteobacteria* and to CH_4_-oxidizing bacteria of the order *Methanosarcinales* [3–5]. Bacteria affiliated within *Gammaproteobacteria* and *Epsilonproteobacteria* are dominant in 4143-1 chimney at the Fuca Ridge hydrothermal vent [14]. Among the most active and abundant microorganisms in Guaymas Basin plumes are sulfur-oxidizing bacteria of the SUP05 group of *Gammaproteobacteria* [15,16]. The most prevalent microorganisms in the *A* *. pompejana* episymbiont community belonged to *Epsilonproteobacteria* [6]. Previous studies showed that chemolithoautotrophs from *Epsilonproteobacteria* and *Gammaproteobacteria* are predominant primary producers in shallow-sea hydrothermal systems [13,17]. However, the 16S rRNA gene survey offered only limited information on the biogeographic patterns of microbial consortia within an environment. Functional genes rather than species may be the appropriate parameter for understanding biological patterns of bacterial communities [18]. Functional gene analyses showed that species belonging to *Gammaproteobacteria* and *Epsilonproteobacteria* in deep-sea hydrothermal vent ecosystems have the potential to grow chemoautotrophically through the Calvin-Benson-Bassham (CBB) cycle and the reductive tricarboxylic acid (rTCA) cycle, respectively [5,19]. Such species can also gain energy by oxidizing reduced sulfur compounds or hydrogen [5,19]. For example, the SUP05 group of *Gammaproteobacteria* can oxidize reduced sulfur compounds using the energy of hydrogen oxidation in Guaymas Basin plumes [16]. In addition, microbial populations are metabolically active in deep-sea hydrothermal systems with genes encoding the oxidation of methane and ammonia [15,20]. *Riftia* symbionts might use the rTCA and CBB cycle pathways for carbon fixation, which is possibly an adaptation to the dynamic vent environment [12].

Previous studies conducted functional metagenomic investigations of deep-sea hydrothermal vent systems [5,6,14,21–23]. However, data on the metabolic capacity of microbes in shallow-sea hydrothermal vent systems remain insufficient. This study is the first to describe in detail the functional potential of free-living microbes from the Kueishantao shallow-sea hydrothermal field using high-throughput sequencing technology. Our comparative metagenomic analysis provided insights into the metabolic processes potentially associated with the adaptation of microbial communities into extreme environments.

## Results and Discussion

### Overview of the geochemical context of sampling sites

Compared with deep-sea vents, shallow hydrothermal systems are characterized by the presence of a gas phase and the enrichment of O_2_ [7]. Furthermore, the extensive mixing of thermal fluids with oxygenated seawater generates micro-scale redox gradients within shallow-sea hydrothermal systems, thereby affecting various ecosystems [7]. Thus, sampling sites denoted as G1 (vent) and G2 (surface water immediately over the vent) were selected to represent the distinct environment conditions of the Kueishantao shallow-sea hydrothermal system. Dissolved inorganic carbon, ammonium, and phosphate in the vent were all higher than those in the surface water. Nitrate, nitrite, CH_4_, salinity, and pH were lower at the vent than at the surface water, whereas temperature was higher at the vent than at the surface water (49 °C) (Supplementary Table S1).

### Characteristics of Kueishantao Shallow-Sea Hydrothermal System Metagenomes

After removing artificial replicates, 266,487 (G1) and 299,124 (G2) sequence reads with average read lengths of 348 and 401 bp, respectively, were used for the analysis. Approximately 62.5% and 66.1% of the total predicted proteins (146,873 and 161,383, respectively) from the G1 and G2 metagenomes show matches against the M5NR database of the MetaGenome Rapid Annotation with Subsystem Technology (MG-RAST) server [24], with 84,209 and 101,639 matches to functional categories, respectively. The unassembled sequences were used for comparative metagenomic analysis. The assembly of single reads from G1 and G2 datasets resulted in 41 and 95 contigs (>5 kbp in length), respectively.

### Taxonomic distribution of metagenomic sequences

The taxonomic classification of protein-coding genes was assigned to the IMG annotation source using the best hit classification of MG-RAST [24]. Bacterial sequences dominated both samples with 97.0% (G1) and 98.7% (G2) of all annotated sequences. Meanwhile, a low number of eukaryotic or other sequences were found. Some sequences were related to proteins affiliated with archaeal members of the class *Thermococci*, accounting for 1.8% of annotated sequences in the G1 metagenome. Approximately 1.4% of the total gene sequences were assigned to *Cyanobacteria* in the G2 metagenome.

**Figure 1:**
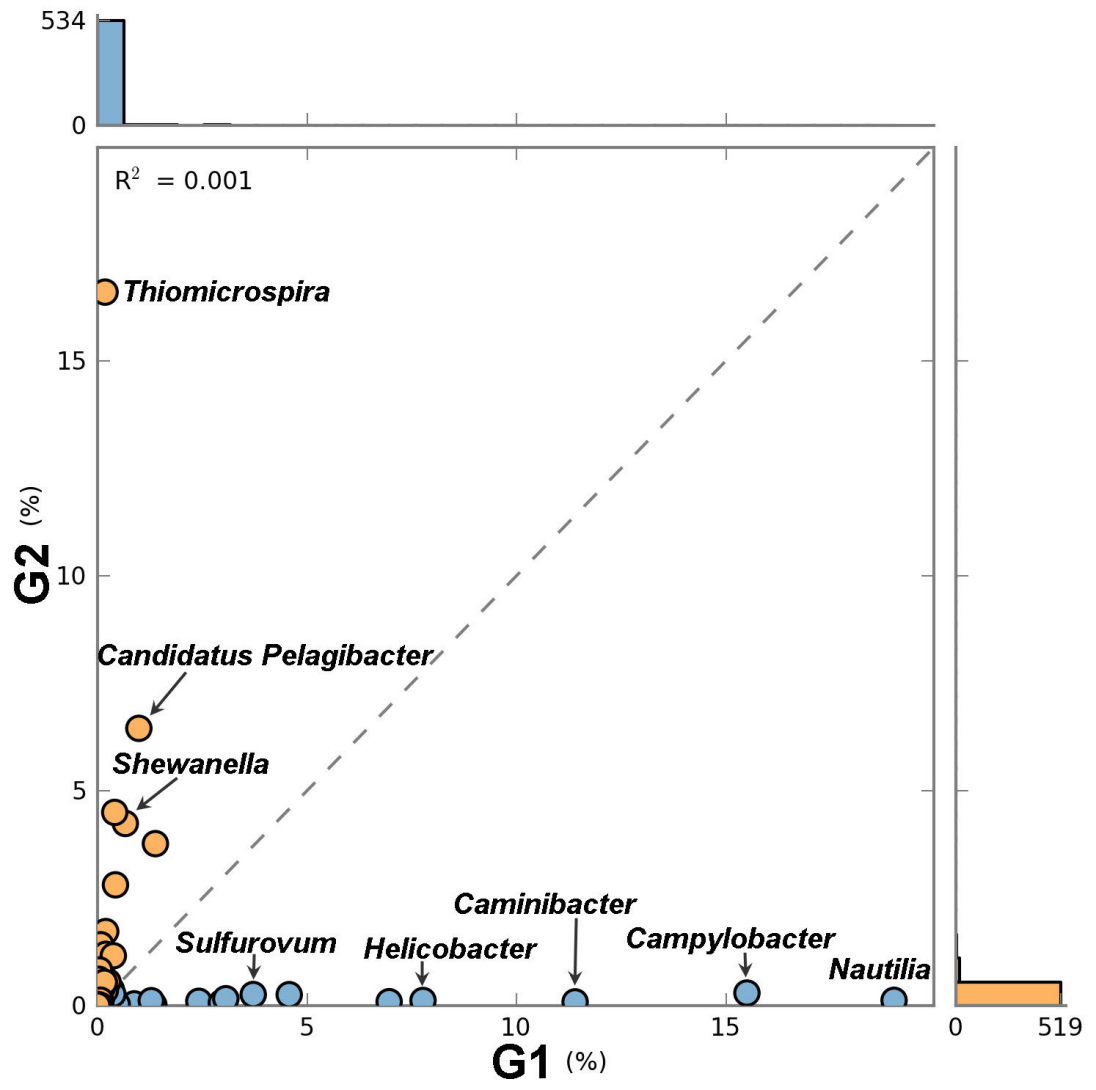
Comparison of the taxonomic profiles at the genus level of two samples from the shallow-sea hydrothermal system. The taxonomic profiles for the vent (G1 colored blue) and surface water immediately above the vent (G2 colored orange) metagenomic datasets were computed using MG-RAST and STAMP v2.0. Corrected P-values (*q*-values) were calculated based on Fisher’s exact test using Storey’s FDR approach. Dots on either side of the dashed trend line were enriched in one of the two samples. Labeled dots at greater distances from the dashed trend line indicate that these subsystems had greater proportional differences (%) between two metagenomes. A filter was applied to remove features with *q* value >0.05.

**Figure 2:**
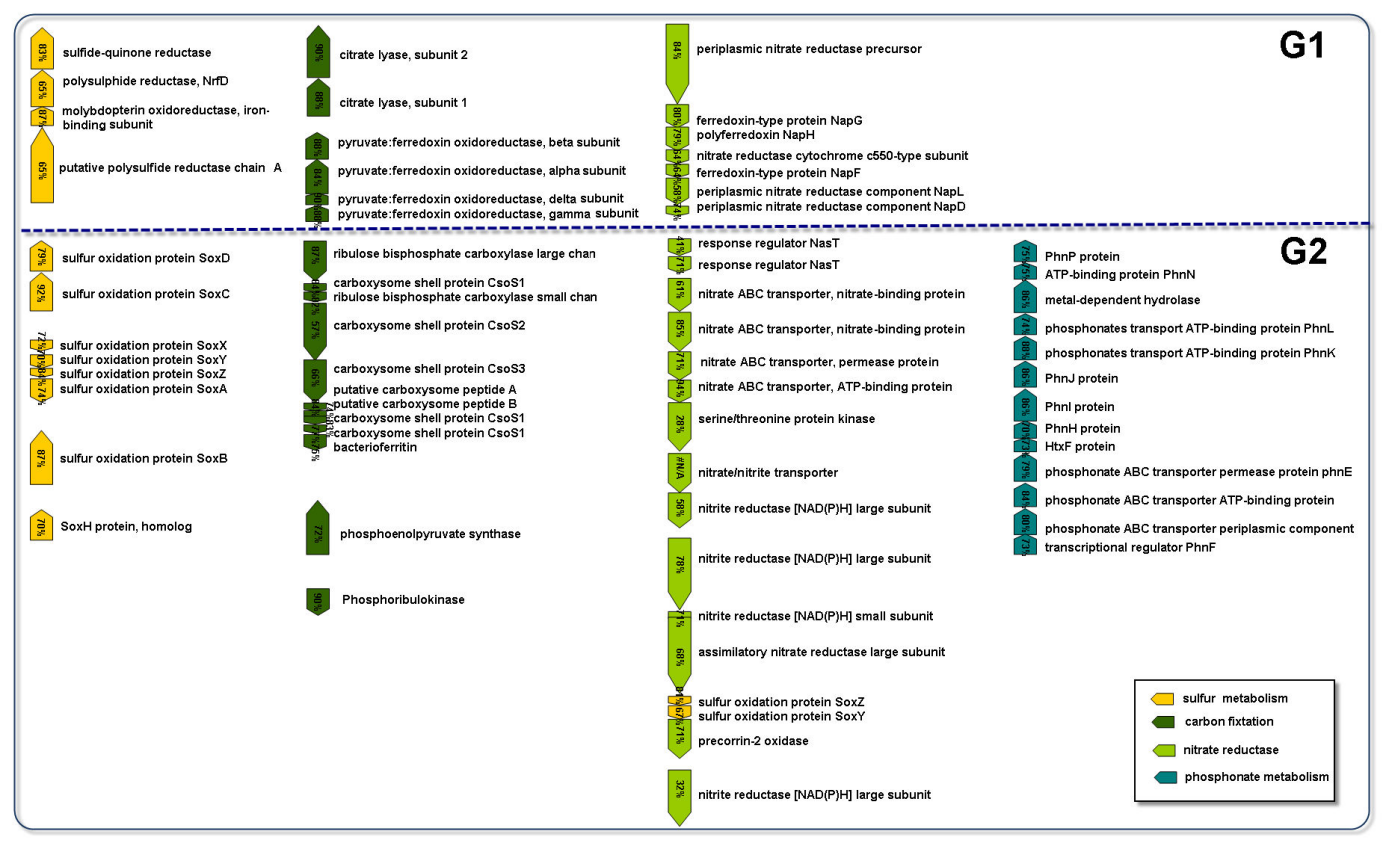
Representative open reading frames encoding carbon fixation, and sulfur, nitrogen and phosphorus utilization-associated functions present in the G1 and the G2 contigs. The % identities of the homologs to the genes from the reference genomes ( *N* *. profundicola* or *C* *. mediatlanticus* as references for the G1 dataset analysis and *T* *. crunogena* as a reference for the G1 dataset analysis) are listed in the colored boxes. #N/A indicates that no best matches to *T* *. crunogena* were found.

The bacterial community was diverse with representatives of more than 40 classes. *Epsilonproteobacteria* was the most dominant class in the vent, accounting for 79.5% of the total assigned sequences of G1. In the surface water, *Gammaproteobacteria* was the most abundant class, accounting for 61.6% of the total sequences of G2, followed by *Alphaproteobacteria* (16.2%) and *Betaproteobacteria* (9.5%). As significant difference in microbial community composition was found between the vent and the surface water. Further statistical analysis indicated that a significant overrepresentation of the sulfur-reducing genera *Nautilia* and *Caminibacter* within the order *Nautiliales* of the class *Epsilonproteobacteria* was observed in the vent and that a significant overrepresentation of the sulfur-oxidizing genus *Thiomicrospira* was found in the surface water (*q* < 0.05, normalized based on metagenome and effect size; Figure 1). Among the sequences assembled from the G1 metagenomic dataset, the majority of the predicted genes show the highest similarity to *Nautilia* *profundicola* strain Am-H [19] or *Caminibacter* *mediatlanticus* TB-2^T^ [25] (Figure 2). Most of the large contigs in the G2 metagenomic dataset contain open reading frames (ORFs) with significant sequence similarity to the completed genome sequence of *Thiomicrospira* *crunogena* XCL-2 [26] (Figure 2). The assembly of these contigs from the metagenomic data provided direct evidence that these communities were dominated by a few populations and may not be very diverse. Bacteria in the *Thiomicrospira* and *Nautiliales* lineages were originally found and isolated from deep-sea hydrothermal vents [19,25,26]. Thus, these organisms could inhabit geochemically distinct habitats. Remarkably, these contigs contained the important genes for ecological implications (Figure 2).

Other significantly different bacterial groups included the genera *Campylobacter* *, Nitratriuptor*, *Sulfurovum* , *Lebetimonas* , *Sulfurimonas* , *Arcobacter* , *Hydrogenimonas* , *Nitratifractor* , *Sulfurospirillum* , and *Helicobacter* in the vent and *Shewanella* *, Vibrio,* *Nitrosococcus* , and *Marinobacter* in the surface water (*q* < 0.05)*. Epsilonproteobacteria* at the deep-sea hydrothermal vents could be considered an evolutionary source of human/animal pathogens [6]. Microbial groups ( *Campylobacter* and *Helicobacter* ) phylogenetically related to important pathogens were found at the shallow-sea hydrothermal vent. *Candidatus* *Pelagibacter* (SAR11 clade) was the most abundant among the *Alphaproteobacteria* species in the surface water. Overall, the taxonomic affiliation of protein sequences displayed a similar pattern of bacterial diversity to taxonomic analysis based on the identified 16S rRNA genes using the M5NRA database (data not shown).

### Metabolic profiling of Kueishantao shallow-sea hydrothermal systems

#### Sulfur metabolism

**Figure 3:**
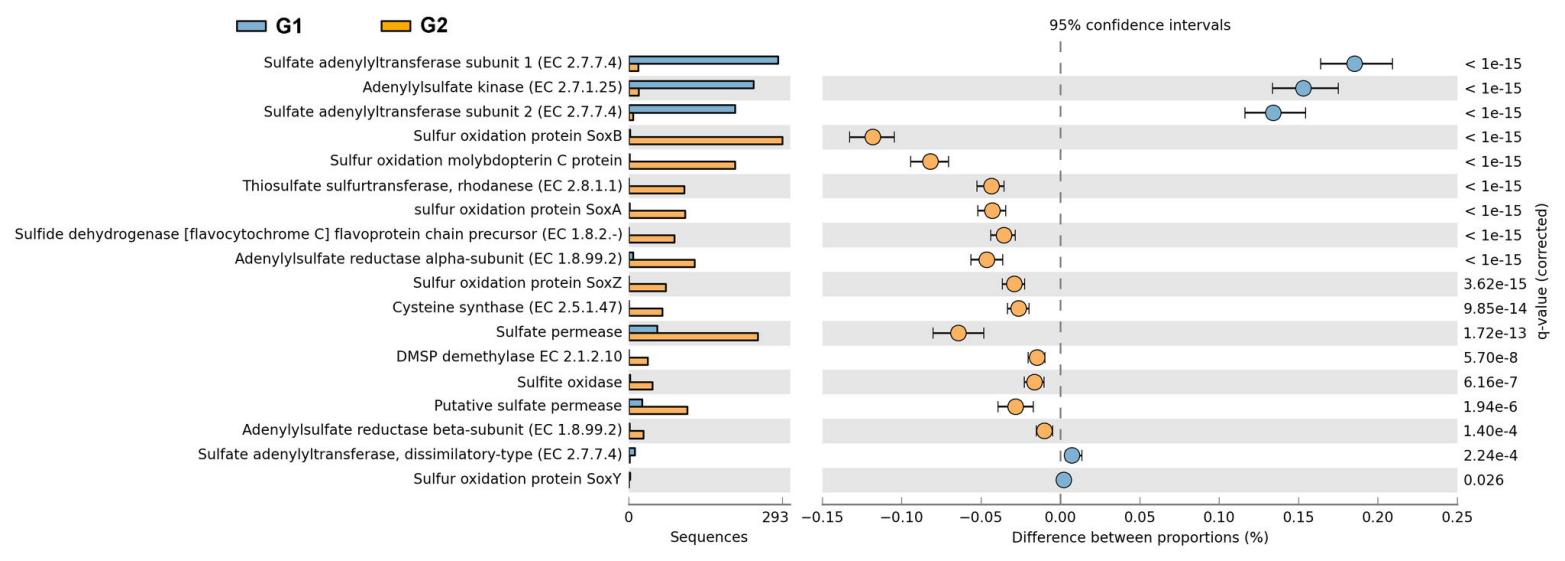
Metagenomic profile comparisons of genes associated with sulfur metabolic pathways determined using STAMP analysis. Positive differences between proportions denote greater abundances in the G1 dataset (blue), whereas negative differences between proportions show greater abundances in the G2 dataset (orange) for the given genes. Corrected P-values (*q*-values) were calculated based on Fisher’s exact test using Storey’s FDR approach. Features with *q* value <0.05 were considered significant and were thus retained.

Major differences in the enrichment of specific gene families within the sulfur pathway were observed (Figure 3). The genes encoding sulfate adenylyltransferase and adenylylsulfate kinase were overrepresented in the vent (Figure 3). However, the sulfate reduction pathway involving dissimilatory sulfite reductase, adenylylsulfate reductase, and sulfate adenylyltransferase is incomplete [27]. However, genes encoding for polysulfide reductase (Psr) were present in a contig from the G1 dataset (Figure 2), resulting the reduction of polysulfide derived from elemental sulfur to sulfide [27]. Thus, sulfur reduction and not sulfate reduction could be performed by the microorganisms in the vent. *Psr* gene sequences in the vent were annotated to species of *Nautilia* or *Caminibacter* . Meanwhile, the contig present in the G1 dataset contained an ORF with an 83% amino acid identity to a sulfide-quinone reductase (Sqr) encoded by *C* *. mediatlanticus* , resulting in the catalysis of the oxidation of sulfide to elemental sulfur (Figure 2) [27]. In addition, 309 and 227 Sqr sequences were detected in the G1 and G2 datasets, respectively, indicating that sulfide oxidation might be an important process in the shallow-sea hydrothermal system. The majority of retrieved *Sqr* gene sequences from the G1 and G2 datasets were assigned to *Nautiliales*-like organisms and *Thiomicrospira* -like organisms, respectively. An overabundance of the other genes associated with sulfur oxidation was present in the G2 dataset (Figure 3). The bacterial community in the surface water possesses genes encoding for key enzymes of three pathways involved in sulfur oxidation [27]: genes encoding adenylylsulfate reductase, sulfide dehydrogenase, and Sox enzyme complex (Figure 3). Most of the *sox* gene sequences were affiliated with *Thiomicrospira* -like organisms. The *sox* operons (*soxXYZA*, *soxCD*, *soxB*, and *soxH*) encoding enzymes for the oxidation of inorganic sulfur compounds [27] were also observed in the G2 contigs (Figure 2). Other genes involved in the oxidation of inorganic sulfur compounds, including sulfite oxidase and thiosulfate sulfurtransferase, were also found in the G2 dataset (Figure 3), which is mainly contributed by sequences related to those in *Gammaproteobacteria*. Therefore, the microbial community in the surface water was probably capable of oxidizing a wide range of reduced sulfur compounds. Moreover, sulfur assimilation in the surface water may involve the metabolism of other sulfur-containing compounds, such as dimethylsulfoniopropionate (DMSP) and cysteine (Figure 3). Genes encoding demethylase for DMSP degradation were found only in the G2 dataset (Figure 3), and the sequences were affiliated with those found in the *Roseobacter* and SAR11 clade.

#### Carbohydrate metabolism and carbon fixation

Approximately 10.5% and 8.8% of the annotated reads from the G1 and G2 metagenomes were categorized within the carbohydrate subsystem. The majority of these sequences were linked to the serine−glyoxylate cycle, central carbohydrate, and CO_2_ fixation subsystems (Supplementary Figure S1). In both metagenomes, the single most abundant component of the carbohydrate subsystems was the serine−glyoxylate cycle followed by the presence of functions involved in the TCA cycle, glycolysis and gluconeogenesis, the CBB cycle, Entner−Doudoroff pathway, pentose phosphate pathway, and pyruvate metabolism (Supplementary Figure S1). However, distinctive differences between the metagenomes in the SEED subsystems were found (*q* < 0.05, Supplementary Figure S1).

**Figure 4:**
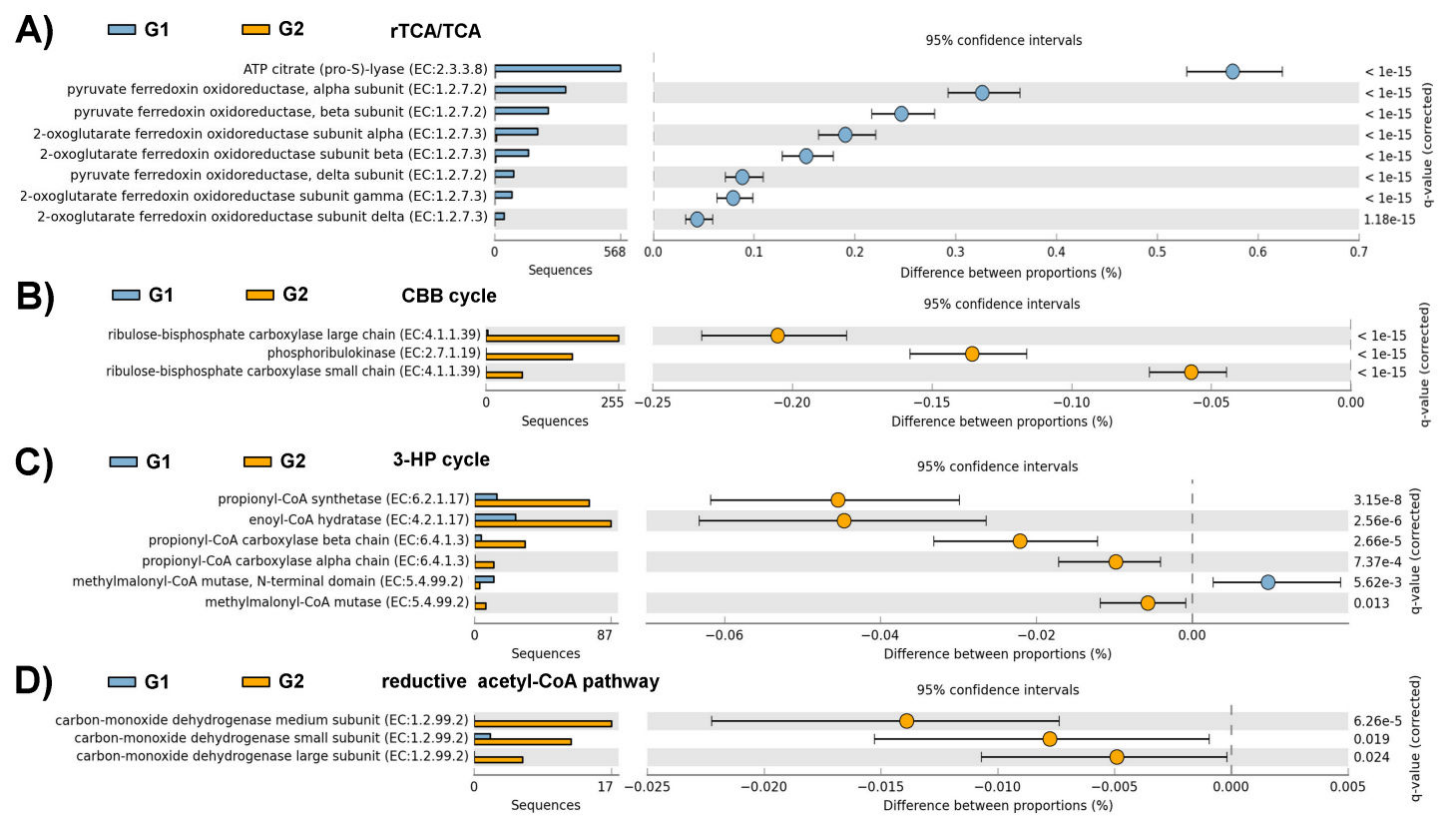
Comparison of genes encoding for key enzymes in the carbon fixation pathways determined using STAMP analysis, including (A) the reverse tricarboxylic acid cycle, (B) the Calvin-Bassham-Benson cycle, (C) the 3-hydroxypropionate cycle, and (D) the reductive acetyl-CoA pathway. Genes encoding enzymes for the G1 (blue) and G2 (orange) datasets were identified based on KEGG functions within the MG-RAST system.

As shown in Figure 4A, the genes for the rTCA cycle [28] were significantly more abundant in the G1 dataset (ATP-dependent citrate lyase, pyruvate: ferredoxin oxidoreductase, and 2-oxoglutarate: ferredoxin oxidoreductase) than in the G2 dataset (*q* < 0.05). This result suggested that microorganisms might utilize the rTCA cycle for CO_2_ fixation in the vent. To identify the likely taxonomic source of specific genes in the metagenomes, all matches obtained by MG-RAST could BLASTX against the National Center for Biotechnology Information (NCBI) nr database. The majority of the annotated sequences involved in the rTCA cycle were related to proteins affiliated with the order *Nautiliales*. Genes involved in CO_2_ uptake (carboxysome) and photorespiration (oxidative C_2_ cycle) were overrepresented in the G2 dataset compared with the G1 dataset (*q* < 0.05, Supplementary Figure S1). This finding suggested that microbes in the surface water possessed a distinguishable carbon fixation pathway. Genes encoding ribulose-1,5-bisphosphate carboxylase (RuBisCO) and phosphoribulokinase that mediate the CBB cycle [28] were enriched in the G2 dataset (Figure 4B). Moreover, one contig in the G2 metagenomic dataset contained a carboxysome operon, including genes for RuBisCO, carboxysome shell proteins, and carbonic anhydrase (Figure 2). Carboxysomes are metabolic modules that enhance the fixation of CO_2_ by RuBisCO [29]. An ORF that is highly similar to the phosphoribulokinase of *T* *. crunogena* was found in the G1 contigs (Figure 2). Genes involved in the CBB cycle in the G1 dataset were mainly affiliated to sequences from *Thiomicrospira* . Thus, organisms in the system might utilize the rTCA and CBB cycles for CO_2_ fixation, similar to what is observed for deep-sea vents [4–6]. Microbial communities in the surface water might have possessed two other CO_2_ fixation pathways, namely, the 3-hydroxypropionate cycle and the reductive acetyl-coenzyme-A pathway [28]. This finding may be attributed to the presence of genes encoding key enzymes involved in these pathways [Figure 4(C) and 4(D)].

#### Nitrogen metabolism

Genes encoding nitrate reductase and the nitrite reductase operon were found in the G2 contig of *T* *. crunogena* (Figure 2), but the nitrate/nitrite transporter was absent in the genome. Homologs of nitrite reductase sequences were identified in the G1 metagenomic dataset. Genes encoding periplasmic nitrate reductase and dissimilatory nitrite reductase were more prevalent in the G1 dataset than in the G2 dataset, especially within the members of *Epsilonproteobacteria*. Genes encoding nitrate reductase and ferredoxin-nitrite reductase were enriched in the G2 dataset (*q* < 0.05, Supplementary Figure S2) and were affiliated with *Thiomicrospira* -like organisms. These data suggested that the microbes in both sites might utilize nitrate as their electron acceptor or nitrogen source. Furthermore, the enrichment of genes encoding hydroxylamine reductase in the G2 dataset suggested that nitrate might be reduced through a nitrate assimilation pathway with hydroxylamine as a key intermediate [30] (Supplementary Figure S2). The gene encoding for ammonia monooxygenase, a key enzyme for ammonia oxidation [20], was not detected in either metagenome.

#### Phosphorus metabolism

Both metagenomes contained high-affinity inorganic phosphate transporters (PstB and PstC). However, the significant enrichment of the low-affinity inorganic phosphate transporter was mostly found in the G1 dataset (*q* < 0.05, Supplementary Figure S3). The phosphonate operon encoding proteins for organic phosphate utilization in *T* *. crunogena* was syntenic with one contig in the G2 dataset (Supplementary Figure S3). The overrepresented *Phn* genes encoding proteins for phosphonate utilization in the surface water [31] might enhance bacterial phosphorus uptake (Supplementary Figure S3).

#### Energy resource

As shown in Figure 2, various metagenomic sequences obtained from shallow-sea hydrothermal systems were highly similar to the genome of the isolates from deep-sea hydrothermal systems [19,25,26]. Based on the identified genes, the dominant organisms in the shallow-sea hydrothermal system near Kueishantao Island with a geochemically dynamic environment can perform different types of metabolism using various abundant potential electron donors (H2, elemental sulfur) and acceptors (elemental sulfur, CO_2_, nitrate, O_2_) as well as potentially lethal levels of heavy metals, such as Fe. Chemoautotrophic bacteria inhabiting the Lost City chimneys [5] and *A* *. pompejana* [6] can obtain their metabolic energy by catalyzing the oxidation and reduction of sulfur, respectively. Similar sulfur reduction or oxidation pathways were found in the Kueishantao shallow-sea hydrothermal field. Under reducing conditions in the vent, both sulfur and polysulfides can serve as terminal electron acceptors [27]. One contig in the G1 dataset appeared to contain a complete Ni–Fe hydrogenase operon similar to the one in *N* *. profundicola* , enabling bacteria to use H_2_ as an energy source in the vents [32]. Bacteria in the surface water can potentially obtain their energy by oxidizing reduced sulfur compounds in the presence of *sox* genes. In general, the oxidation of reduced sulfur compounds can be coupled to the reduction of electron acceptors, including oxygen and nitrate [27]. Furthermore, one contig in the G2 dataset contained genes encoding for cbb3-type cytochrome c oxidases with the potential to mediate aerobic respiration or to act as an electron acceptor even under oxygen-limited conditions [33].

Our 16S rRNA clone data suggested that a shift readily occurred in the predominant microbial population from *Epsilonproteobacteria* to *Gammaproteobacteria* across the redox gradients from the vents to the surface water (data not shown). The co-occurrence of sulfur-oxidizing and sulfur-reducing activities could couple their distinct biogeochemical processes based on reciprocal exchange of sulfur compounds and thereby increase the overall energy efficiency of the shallow-sea hydrothermal community even under a relative lack of sulfide conditions. This result is in agreement with the results from the deep-sea hydrothermal microbial symbiosis [6].

According to 16S rRNA gene analysis, archaea related to methanogens and methanotrophs contribute to a much greater percentage of the total sequences in the deep-sea hydrothermal vent than in the shallow-sea hydrothermal system [13,17]. Deep-sea CH_4_-rich hydrothermal fluids support the growth of large methanogenic and methanotrophic communities, such as those in the Lost City chimneys [5,11]. No genes encoding the key enzymes in methanogenesis/methanotrophy (*mcrA* and *pmoA*) [15] were found in the shallow-sea hydrothermal system. This result suggested that these microbial processes are not dominant, although their fluids contained abundant CH_4_.

Overall, the metabolic profiles of the chemoautotrophic members in the Kueishantao shallow-sea hydrothermal field were similar to those in deep-sea hydrothermal fields, with sulfur metabolism and carbon fixation being of particular importance. Varying concentrations of chlorophyll *a* in the Kueishantao shallow-sea hydrothermal field indicated that phytoplankton likely contributed to carbon fixation using light as the alternative energy source (Supplementary Table S1). Genes encoding for a light-driven proton pump (proteorhodopsin) involving phototrophy were detected in the surface water and were related to those in the SAR11 clade (details in annotation tables for a metagenome at the MG-RAST Website). Furthermore, the higher concentrations of dissolved organic carbon in the deep-sea hydrothermal fields provided energy support for the heterotrophic activity in the Kueishantao shallow-sea hydrothermal system (Supplementary Table S1). The taxes associated with heterotrophy are more prevalent in our metagenomes than those typically found in deep-sea hydrothermal systems [5,6].

### Stress genes

Genes associated with stress response, resistance, and virulence contributed by different bacterial groups were identified in the shallow-sea hydrothermal system (Supplementary Figure S4). Among these genes, the G1 dataset was overrepresented in some subsystems, involving bacterial hemoglobins, periplasmic stress, acid resistance mechanisms, pathogenicity, multidrug resistance efflux pump, and arsenic resistance (*q* < 0.05, Supplementary Figure S4). Genes assigned to oxidative stress and cobalt−zinc−cadmium resistance subsystems, heat shock dnaK gene cluster, and copper homeostasis were statistically overrepresented in the G2 dataset (*q* < 0.05, Supplementary Figure S4). *Thiomicrospira* species were identified as important sources of these genes.

### Functional comparisons of the hydrothermal system metagenomic datasets

**Figure 5:**
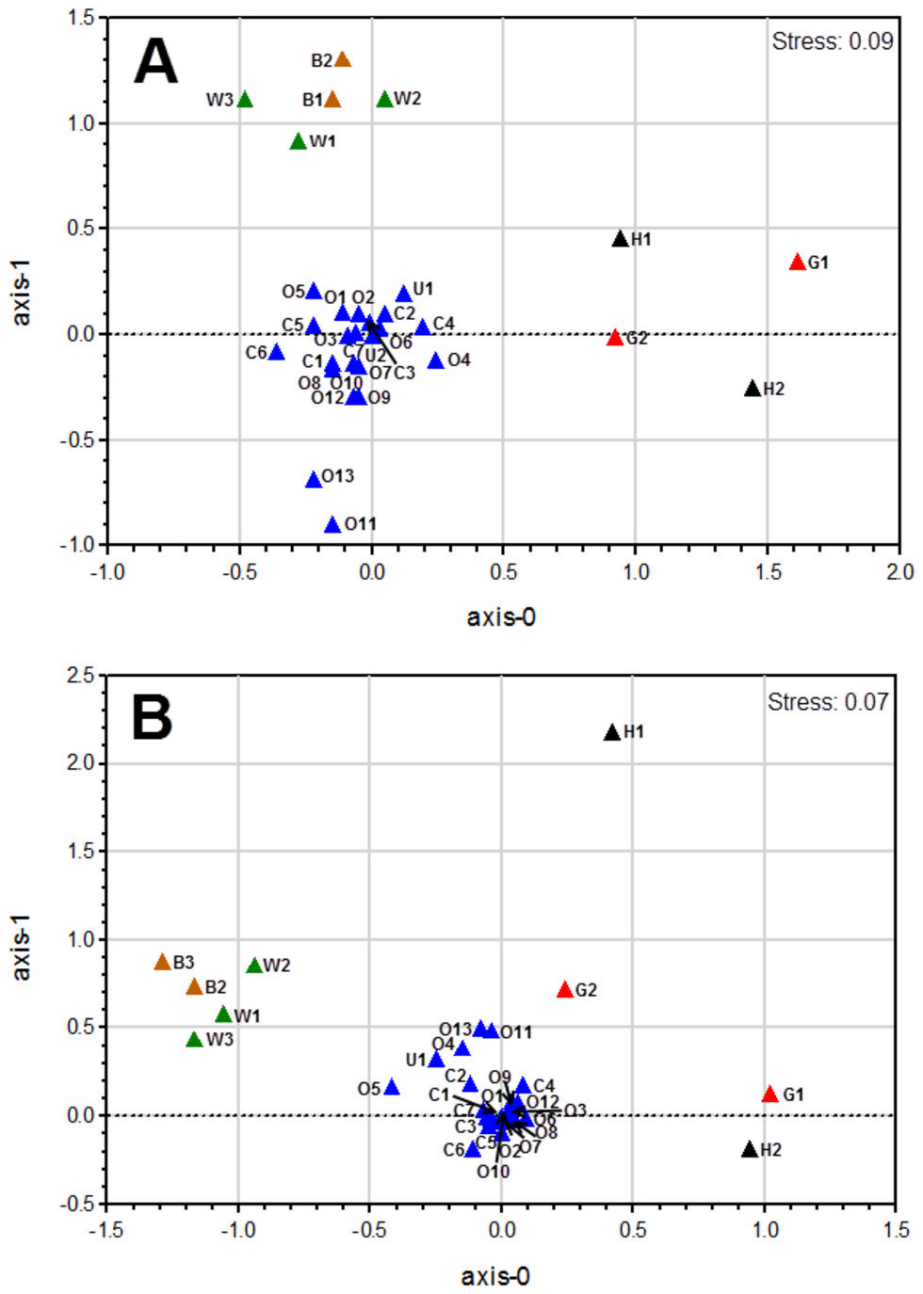
Multidimensional scaling (MDS) plots of samples using Bray–Curtis similarity according to (A) SEED subsystem and (B) Clusters of Orthologous Groups of protein functional annotations. Color represents different sampling areas and each habitat label type (n) is indicated [G: shallow-sea hydrothermal field (red), H: deep-sea hydrothermal field (black), O: open sea (blue), C: coastal and estuary (blue), U: other habitats in common ocean (blue), B: biofilm (orange), W: whale biofilm (green). For details, see Supplementary Table S2]. Samples from each of the respective environments clustered together based on their functional profile. The stress values are reported in the top right corner of the figures and represent the goodness-of-fit.

Based on the relative abundance of Clusters of Orthologous Group (COG) categories and SEED subsystems, multidimensional scaling (MDS) plots showed that most of the samples from the open ocean to the coast, as well as biofilm samples (Supplementary Table S2), clustered closely together at the functional level, apart from hydrothermal field samples (H1: Lost City chimneys [5], H2: *A* *. pompejana* episymbiont community [6]) (Figure 5). This result suggested that hydrothermal systems had functional community profiles distinct from other samples. COG analysis also indicated that the sample from H2 was closely related to the G1 samples on the first ordination axis (Figure 5B). Species belonging to *Epsilonproteobacteria* were also predominant [6].

The two-way functional comparisons revealed some dissimilarity in the SEED subsystems of the samples (Supplementary Table S3). A relatively higher abundance of cobalt−zinc−cadmium resistance genes and virulence genes (e.g. phage integration and excision genes) were found in the deep-sea vents (H1, H2) than in the shallow-sea vent (G1). The genes with significant overrepresentation in the shallow-sea vent relative to deep-sea vents included those for bacterial mobility and chemotaxis (*q* < 0.05). *Campylobacter* and *Helicobacter* species accounted for most of these genes (details in annotation tables for a metagenome at the MG-RAST Website). In submerged environments, motility and chemotaxis-related functions enable bacteria to respond rapidly to environmental changes [34].

**Figure 6:**
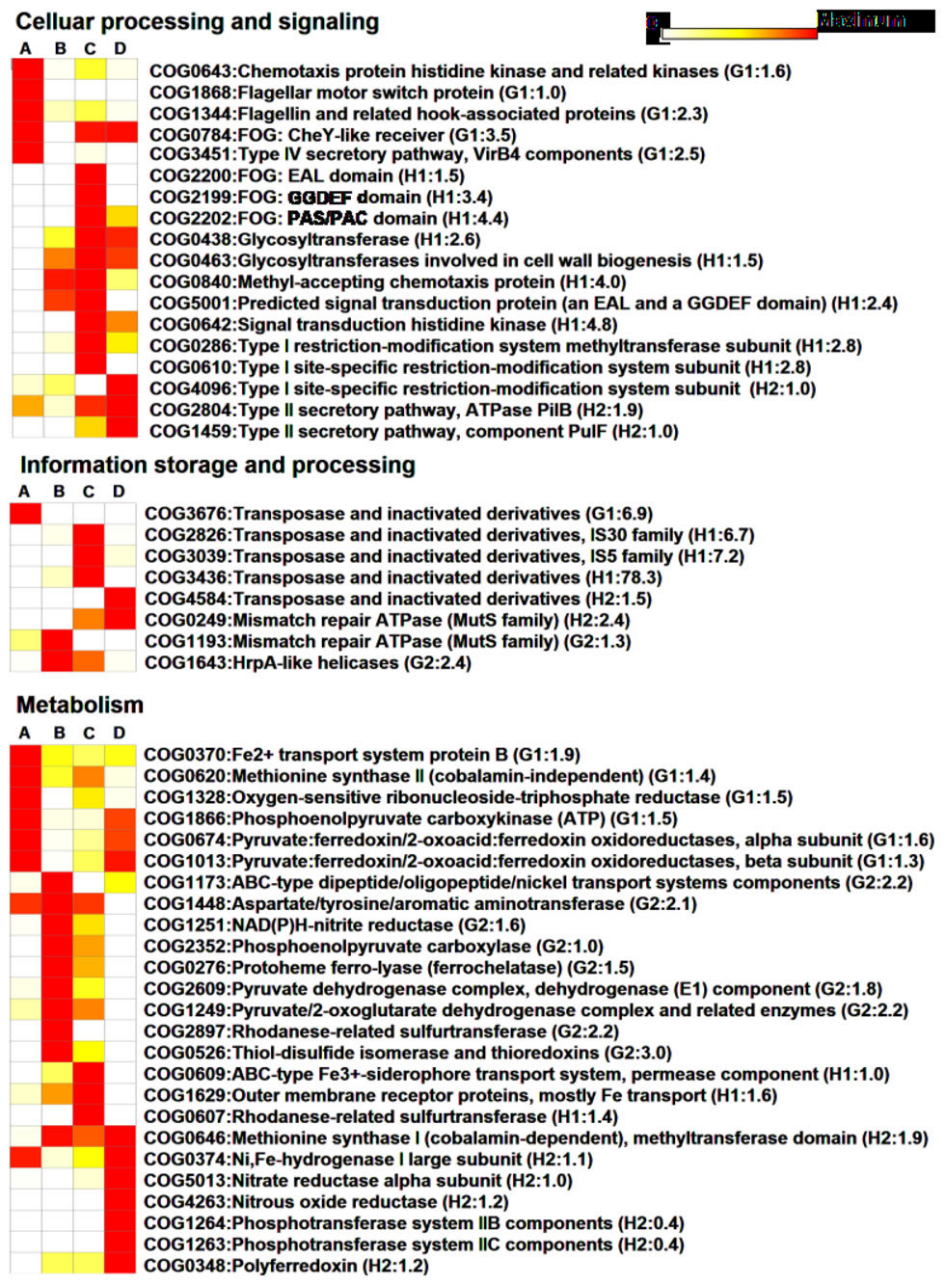
Selected COGs in the order of their contribution to the difference between two samples as assessed using similarity percentage analysis. Each row represents the relative frequency of genes among samples (A: G1, B: G2, C: H1, D: H2). The value of normalized abundance of genes (relative to the single-copy gene *RecA*) is assigned with a color relative to the maximum value among all comparisons of each COG, from white to red. The colors represent 0 (white) to maximum (MAX, red) increments of MAX/10. The higher values indicate greater gene abundance. COG descriptions are listed along the row. Among these COG categories, the maximum *RecA*-normalized gene abundance is shown in a bracket.

Similarity percentage (SIMPER) analysis further revealed the main contributors to the dissimilarity between the two datasets (Figure 6). More genes associated with motility (COG1868, COG1344) and chemotaxis (COG0643) were identified in the G1 dataset than in the other datasets (Figure 6). Type IV secretion systems were more abundant in G1 than in the other sites that were used for both DNA and protein transfer between bacteria or between bacteria and hosts [35] (Figure 6). The type IV secretion system and transposases have potentially important functions in the horizontal gene transfer for deep-sea microbes [21]. Among the vents, H1 contained the most genes encoding transposases (COG2826, COG3039, COG3436) (Figure 6). The G1 dataset also contained abundant genes encoding transposases (COG3676). This finding suggested that the horizontal gene transfer in the shallow-sea hydrothermal fields and in the deep-sea vent chimney biosphere might be a common occurrence. Two-component systems are commonly used by prokaryotes to sense and respond to changing environmental conditions [36]. As shown in Figure 6, the shallow-sea hydrothermal system contains genes for signal transduction EAL domain proteins (COG5001) and FOG: CheY-like receiver protein (COG0784) through which microorganisms can respond to chemical composition changes outside the cell [36]. The H1 metagenome was enriched in genes associated with signal transduction functions, particularly the EAL domain protein (COG5001) and the FOG: GGDEF and FOG: EAL domain protein (COG2199, COG2200) sequences (Figure 6). These domains are involved in the regulation of bacterial growth and survival phenotypes such as the biofilm [36]. In addition, genes encoding PAS/PAC domain proteins (COG2202) were found in the H1 dataset; these genes function as internal sensors of redox potential and oxygen [37]. The H2 metagenome had more genes encoding MutS proteins (COG0249) for DNA mismatch repair than the other metagenomes (Figure 6).

Genes encoding the key enzyme for rTCA cycle-pyruvate: ferredoxin oxidoreductase (COG0674, COG1013) were more abundant in the shallow-sea vent than in the deep-sea vent (Figure 6), reflecting a possible adaptation of the organisms to the high CO_2_ present within the shallow-sea hydrothermal system. In addition, greater abundance of phosphoenolpyruvate carboxykinase and phosphoenolpyruvate carboxylase (COG1866, COG2352) was found in the shallow-sea vent than in the deep-sea vent (Figure 6). These enzymes elicit anaplerotic activities by replenishing oxaloacetate to the rTCA/TCA cycle [38]. Genes encoding nitrate reductase (COG5013) and nitric oxide reductase (COG4263) involved in nitrogen metabolism were enriched in the H2 dataset. Ni–Fe hydrogenases (COG0374) for H_2_ oxidation were observed primarily in the G1 and H2 datasets. Glycosyltransferases (COG0438, COG0436) and the phosphotransferase system (COG1263, COG1264) were more abundant in the H2 metagenome than in the G1 metagenome and were involved in catalyzing the attachment of sugars to protein and transporting sugar [39], respectively. Moreover, glycosyltransferases might have important functions in biofilm development in several bacteria [40]. Vent fluids from the shallow-sea hydrothermal system contain extremely low concentrations of trace metals, such as Fe [7]. Transporter for Fe^3+^ (COG1629, COG0609) genes were enriched in the H1 dataset, whereas Fe^2+^ transporter genes (COG0370) were identified in all datasets (Figure 6).

All these functions could contribute to community adaptations to the hydrothermal environment. The functional genes varied among the metagenomes. Moreover, the microbial communities in the shallow-sea hydrothermal system contained some apparent functional features (Figure 6) that are not found in other environments [5,6]. However, special functional categories, such as transposase and carbon fixation, were found in several datasets. This result indicated that a core of genes was shared by the vent-associated bacterial community.

## Materials and Methods

### Sampling

Samples were collected in July and August 2010 from two depths in the hydrothermal vent system (121°57′E, 24°50′N): one located in the vent (G1: 17.2 m) and one immediately located above the vent (G2: 0 m). The vents were identified by scuba divers, and their positions were located by the global positioning system. Geochemical features of each sampling site were obtained, as shown in Supplementary Table S1. An enriched content of elemental sulfur was observed during sampling. All necessary permits were obtained for the described field studies. Two permits were required and obtained, one from the Coast Guard Administration of Taiwan and the other from the Fisheries Management Office of the Yilan County, Taiwan.

A total of 20 L of seawater was filtered onto 3 µm of GF/C filters (PALL Corporation) and then collected in 0.22 µm of Sterivex filter units (millipore) [41]. After filtration, the Sterivex units were filled with 1.8 mL of lysis buffer (50 mM Tris-HCl, 40 mM EDTA, 0.75 M sucrose, pH 8.3) and were stored at −80 °C until DNA extraction.

### DNA extraction and sequencing

Nucleic acid extraction was performed on a Sterivex filter unit as previously described [42]. Briefly, the samples were added with 100 µL of lysozyme (final concentration of 6.25 mg/mL) and RNase A (final concentration of 100 µg/mL) and then incubated for 1 h. Subsequently, the samples were added with 100 µL of Proteinase K (QIAGEN) and 100 µL of 20% (w/v) sodium dodecyl sulfate and then incubated at 55 °C for 2 h in a hybridization oven. After separation of this lysate, nucleic acids were extracted with an equal volume of phenol: chloroform: isoamyl alcohol (25:24:1, v: v: v). DNA samples were further concentrated by centrifugation (3500×g) with Amicon Ultra-15 30K Centrifugal Filter Units (millipore) and washed several times in TE buffer (pH 8.0). DNA concentration was estimated using a NanoDrop 2000 spectrophotometer (Thermo, Fisher, USA) and analyzed through gel electrophoresis. Approximately, 5 µg of DNA per sample was sent for pyrosequencing to the Chinese National Human Genome Center (Shanghai, China). Shotgun sequencing runs were performed on libraries prepared from environmental samples of community DNA using the 454 GS FLX Titanium protocols.

### Metagenome sequencing, assembly, and annotation

The 454 sequencing reads were filtered using an in-house developed program to remove low-quality reads. A total of ~92.82 (118.79) Mbp (G1) and ~120.22 (172.79) Mbp (G2) of unique sequence data was generated from the G1 and G2 samples. Raw sequencing reads from both datasets were submitted to the MG-RAST server (version 3.0) for gene annotation (http://metagenomics.anl.gov/) [24]. The artificially created duplicate reads were removed automatically by MG-RAST [24]. The putative ORFs were identified, and their corresponding protein sequences were searched with BLAST against the M5NR non-redundant protein database in the MG-RAST (an E-value cutoff of less than 1 × 10^-3^). M5NR is an integration of many sequence databases [including the NCBI GenBank, COG, Kyoto Encyclopedia of Genes and Genomes (KEGG), and SEED] into a single, searchable database [24]. Raw sequencing reads were assembled into contigs employing de novo assembler software Newbler (454 Life Sciences, Roche Applied Sciences, Branford, CT, USA). Newbler could be superior to some assemblers for merging 454 sequencing reads into longer contig, but the amount of contigs produced by Newbler was possibly less [43,44].

### Comparative metagenome analyses

Taxonomic and functional profiles within MG-RAST (hits to IMG, M5NRA, SEED, COG, and KEGG databases) were extracted (an E-value cutoff of less than 1 × 10^-5^ and a minimum read length of 50 bp) to compare functional attributes across metagenomes. For all subsequent analyses, gene counts were normalized against the total number of hits in their respective databases to remove bias in different sequencing efforts as described previously [45,46]. To explore functional differences between two metagenomic datasets, Statistical Analysis of Metagenomic Profiles (STAMP) v2.0 software package [47] was employed to test for significant differences in both taxonomic and functional distribution between metagenomes. Statistical significance of differences between samples (*q* value) was assessed using the two-sided Fisher’s exact test with Storey’s false discovery rate method of multiple test correction within STAMP [47]. The confidence intervals were determined using the Newcombe−Wilson method. Features with a *q* value of <0.05 were deemed significant.

To extend comparative metagenomic analyses, other marine environmental metagenomic datasets publicly available in MG-RAST were selected (Supplementary Table S2). This E-value (1 × 10^-3^) was used for the comparative analyses of metagenomic datasets with different read lengths [45,46]. The abundance of COG categories or SEED subsystems per metagenome was transformed using square root, and Bray−Curtis similarities were calculated on the data matrices. Non-metric MDS was used to determine the similarity among datasets with the PRIMER-E ecological software package [48,49]. The similarities are presented in a multidimensional space by plotting more similar samples closer together using the Ginkgo software [50]. SIMPER analysis in PRIMER-E was used to determine the similarity or difference between the COG abundance distributions among representative metagenomic datasets from the hydrothermal fields [49]. To remove the bias of average genome size on the sampling of gene from a given metagenomic community, the abundance of gene in each COG was normalized against the number of single-copy *RecA* gene per metagenome [51,52]. The top 50 COG values representing relatively more contributions to the differences between the two samples were selected for heatmap visualization.

### Data availability

The sequence data are available under “Kueishantao metagenomes project” in the MG-RAST database (http://metagenomics.anl.gov/) (ID 4487624.3 for vent, and ID 4487625.3 for surface water above the vent). All individual sequence reads have been deposited at the NCBI Short Read Archive (SRA) under the accessions SRX202013 for the vent and SRX202014 for surface water above the vent datasets.

## Supporting Information

Figure S1 **Comparisons of SEED carbohydrate subsystem for the G1 (blue) and G2 (orange) datasets determined using STAMP analysis.** Classification of a pathway is based on SEED subsystem hierarchy 3 of the MG-RAST.
(TIF)

Figure S2 **Metagenomic profile comparisons of genes involved in nitrogen metabolism for the G1 (blue) and G2 (orange) datasets determined using STAMP analysis.** Enzyme identification was based on KEGG functions within the MG-RAST system.
(TIF)

Figure S3 **Metagenomic profile comparisons of genes associated with phosphorus utilization pathways for the G1 (blue) and G2 (orange) datasets determined using STAMP analysis.** Enzyme identification was based on KEGG functions within the MG-RAST system.
(TIF)

Figure S4 **Comparison of genes associated with stress and virulence determined using STAMP analysis.** Gene identifications were based on subsystem hierarchy 4 of the MG-RAST system.
(TIF)

Table S1 **Geochemical data of sampling sites.**
(XLS)

Table S2 **Information on publicly available metagenomes used in this study.**
(XLS)

Table S3 **Selected pair-wise comparisons of SEED subsystems for the shallow-sea and deep-sea hydrothermal fields’ metagenomic datasets.**
(XLS)
