# Supplementary figures and images for: Functional Metagenomic Investigations of Microbial Communities in a Shallow-Sea Hydrothermal System
Source: PLoS One. 2013 Aug 6;8(8):e72958. doi: 10.1371/journal.pone.0072958 (PMC3735525; doi:10.1371/journal.pone.0072958)

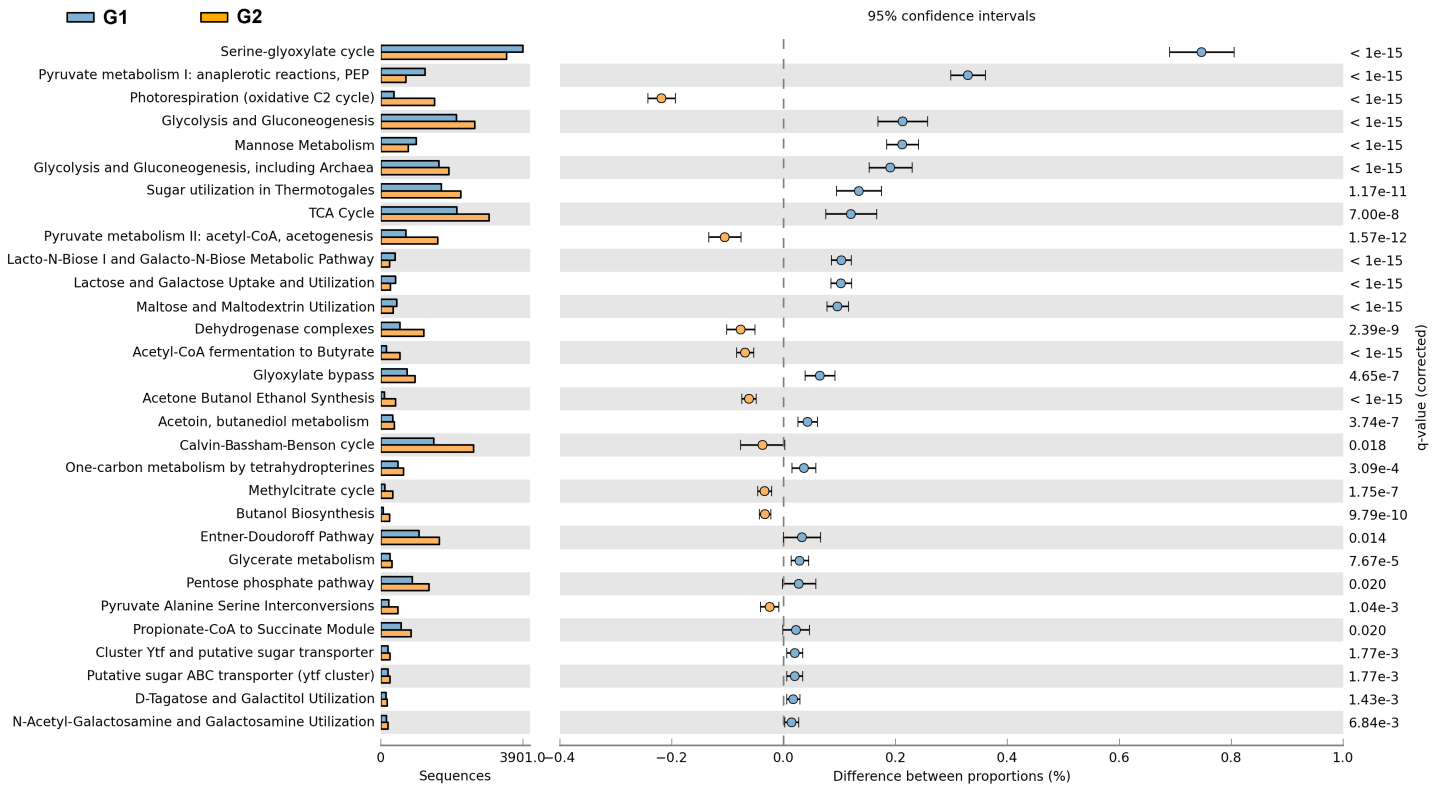

Supplement: Figure S1 — Comparisons of SEED carbohydrate subsystem for the G1 (blue) and G2 (orange) datasets determined using STAMP analysis. Classification of a pathway is based on SEED subsystem hierarchy 3 of the MG-RAST. (TIF) [file pone.0072958.s001.tif]

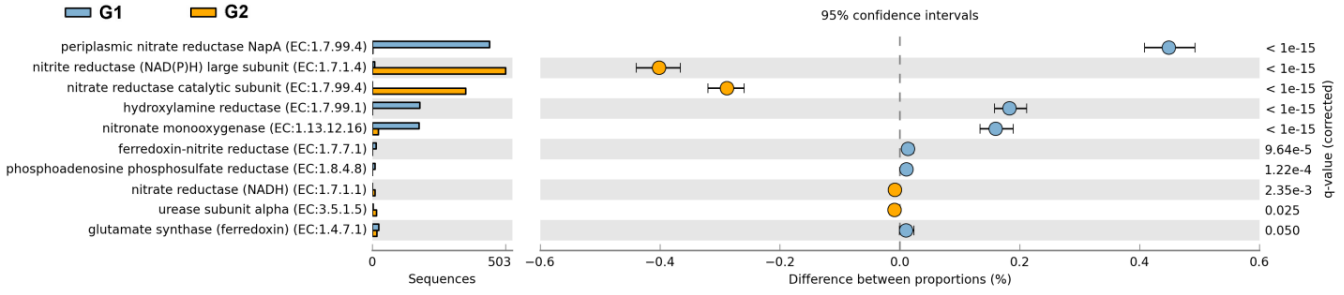

Supplement: Figure S2 — Metagenomic profile comparisons of genes involved in nitrogen metabolism for the G1 (blue) and G2 (orange) datasets determined using STAMP analysis. Enzyme identification was based on KEGG functions within the MG-RAST system. (TIF) [file pone.0072958.s002.tif]

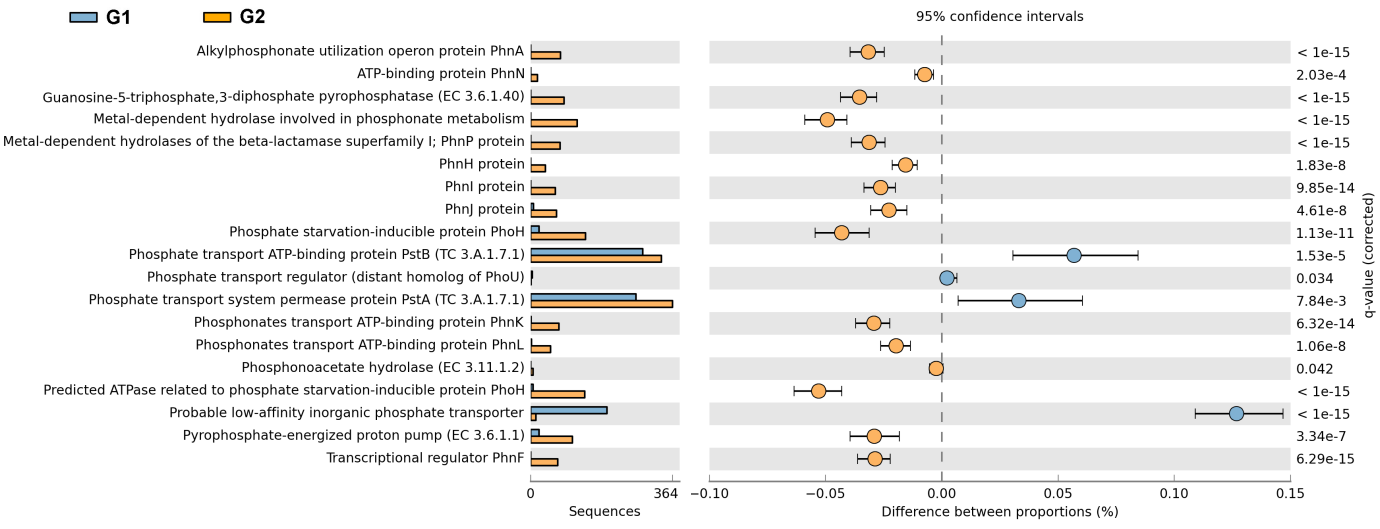

Supplement: Figure S3 — Metagenomic profile comparisons of genes associated with phosphorus utilization pathways for the G1 (blue) and G2 (orange) datasets determined using STAMP analysis. Enzyme identification was based on KEGG functions within the MG-RAST system. (TIF) [file pone.0072958.s003.tif]

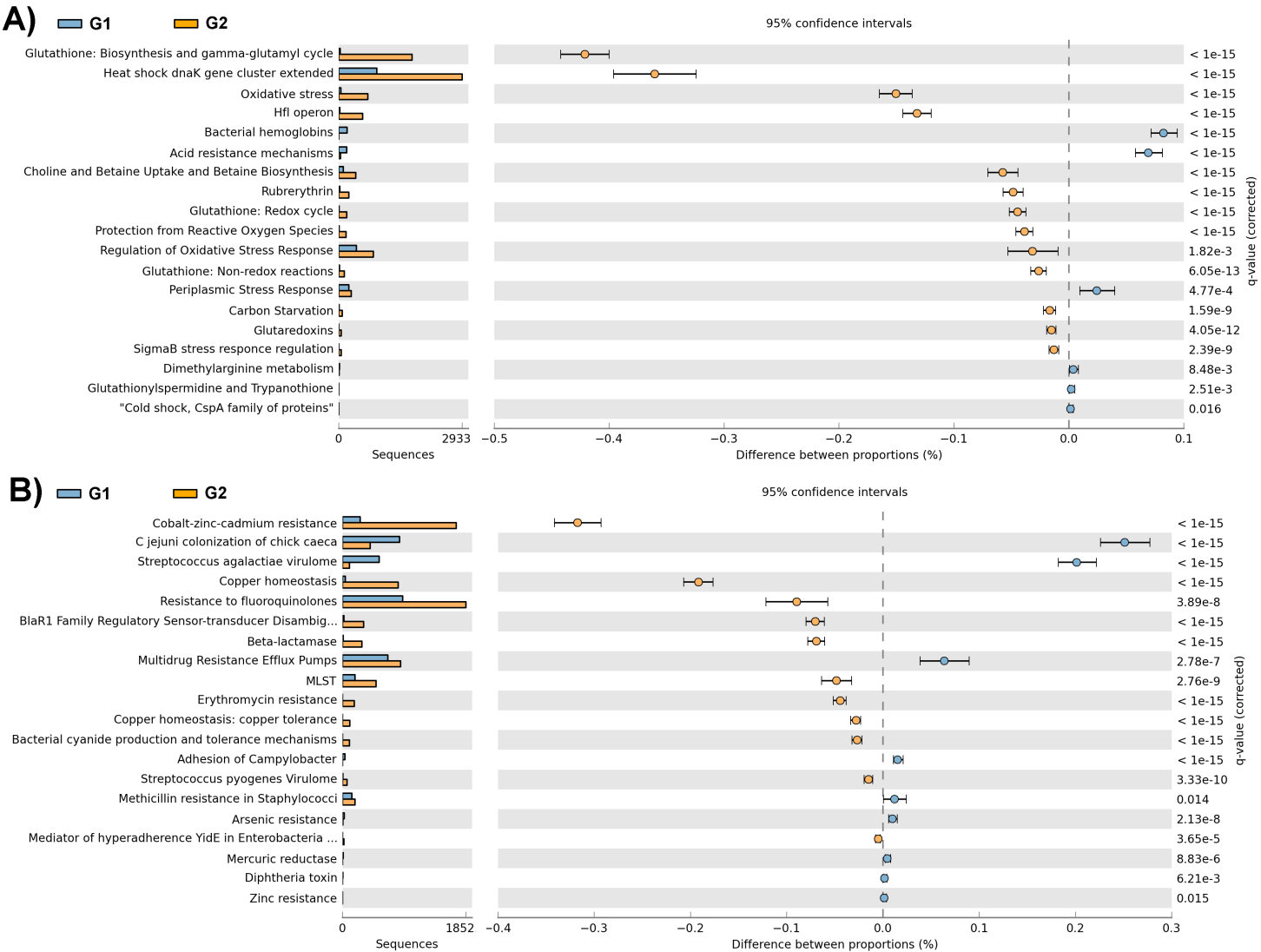

Supplement: Figure S4 — Comparison of genes associated with stress and virulence determined using STAMP analysis. Gene identifications were based on subsystem hierarchy 4 of the MG-RAST system. (TIF) [file pone.0072958.s004.tif]
